# Case Report: Second-line favourable effect of pembrolizumab plus chemotherapy in a patient with metastatic hepatoid adenocarcinoma of the stomach

**DOI:** 10.3389/fphar.2025.1387661

**Published:** 2025-04-01

**Authors:** Yao Tang, Xiaofen Li, Yu Yang

**Affiliations:** ^1^ Department of Medical Oncology, Cancer Center, West China Hospital, Sichuan University, Chengdu, China; ^2^ Division of Abdominal Tumor Multimodality Treatment, Cancer Center, West China Hospital, Sichuan University, Chengdu, China

**Keywords:** hepatoid adenocarcinoma of the stomach(HAS), case report, pembrolizumab, chemotherapy, immunotherapy

## Abstract

Hepatoid adenocarcinoma of the stomach (HAS) is a rare and aggressive malignancy with poor prognosis. HAS is characterized by both adenocarcinoma and hepatocellular carcinoma like differentiation, which often produces alpha-fetoprotein (AFP) and responds poorly to systemic chemotherapy. Herein we report a case of 62-year-old male diagnosed with liver metastatic HAS, who progressed rapidly after first-line cisplatin + fluorouracil chemotherapy combined with pembrolizumab. However, the patient achieved favourable outcome after second-line oxaliplatin + capecitatine chemotherapy combined with pembrolizumab. This case indicated that the chemotherapy regimen might influence the efficacy of immune check-point inhibiors plus chemotherapy.

## Introduction

Hepatoid adenocarcinoma of the stomach (HAS) is a rare subtype of gastric cancer, which only accounts for 0.3%–1% of all gastric cancers (Zhu et al., 2022). HAS is mainly characterized by hepatocyte like differentiation and AFP production in tumor tissue (Gao et al., 2007). The concept of HAS originated from AFP-producing gastric cancer (AFPGC). Since 1970, Bourreille (Bourreille et al., 1970) et al. first reported a case of gastric cancer with liver metastasis accompanied by AFP production, but there was no pathological evidence to support it at that time, so it was only referred to as “AFP-producing gastric cancer”. Due to the rarity and pathological heterogeneity of HAS, it is still not fully understood and often prone to misdiagnosis and missed diagnosis (Zhang et al., 2020).

The prognosis of HAS is extremely poor. Most patients are diagnosed in the advanced stage and have lost the opportunity for surgical resection. The first-line treatment relys on the treatment plan for gastric adenocarcinoma, mainly chemotherapy combined with immunotherapy (Ajani et al., 2022). Herein, we reported a case of liver metastatic HAS patient who progressed rapidly after first-line FP chemotherapy (fluorouracil + cisplatin) plus pembrolizumab. Fortunately, second-line CAPEOX chemotherapy (oxaliplatin + capecitatine) plus pembrolizumab immunotherapy achieved favourable efficacy. The different results obtained by the two regimens may be related to the synergistic effect of oxaliplatin and immunotherapy. Oxaliplatin chemotherapy combined with immunotherapy can stimulate a broader immune response by inducing immunogenic cell death and disrupting immune escape of tumor cells. In addition, oxaliplatin can not only directly kill tumor cells, but also enhance the efficacy of immunotherapy by changing the tumor microenvironment. Oxaliplatin can promote the activation of antigen-presenting cells in various ways, enhance the uptake and presentation of tumor antigens, activate cytotoxic T lymphocytes (CTL), and induce direct cytotoxic reactions to kill residual tumor cells.

## Case presentation

**FIGURE 1:**
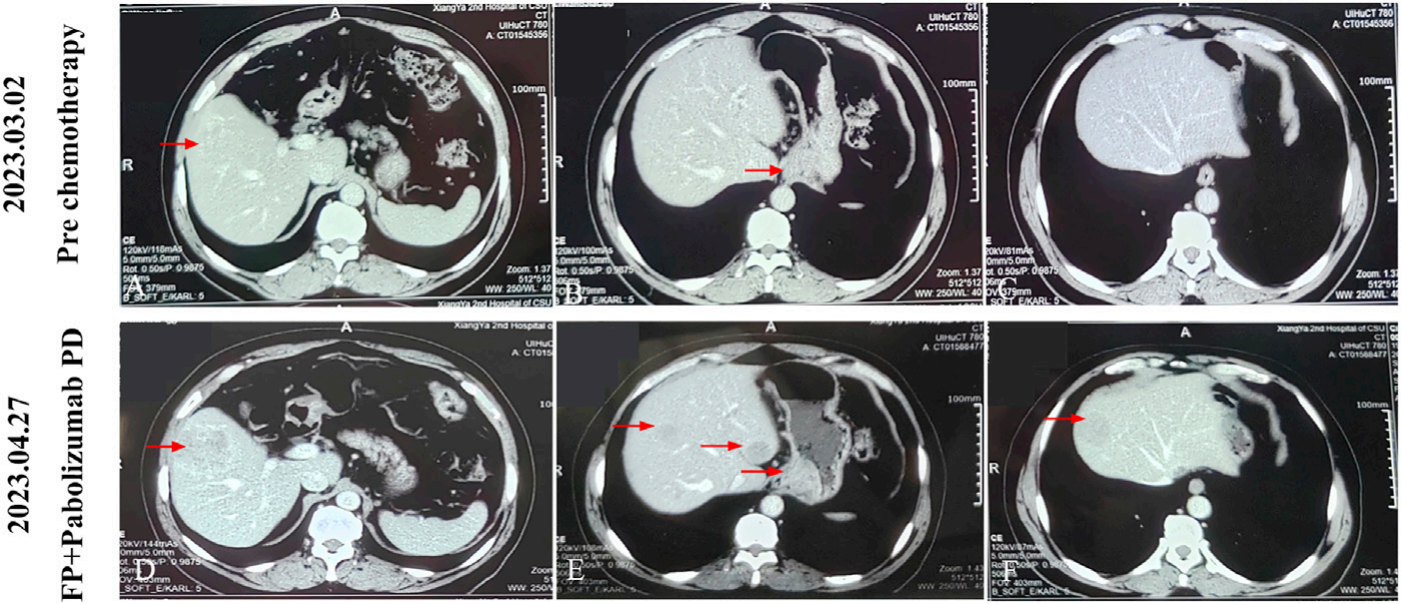
After three cycles of treatment with FP regimen combined with pembrolizumab, the patient’s tumor progressed. **(A-C)** The baseline examination images of the patient, or the images before anti-tumor treatment. **(D-F)** The images of the patient after first-line three cycles of FP regimen combined with pembrolizumab. The red arrow indicates the area where the tumor is located.

**FIGURE 2:**
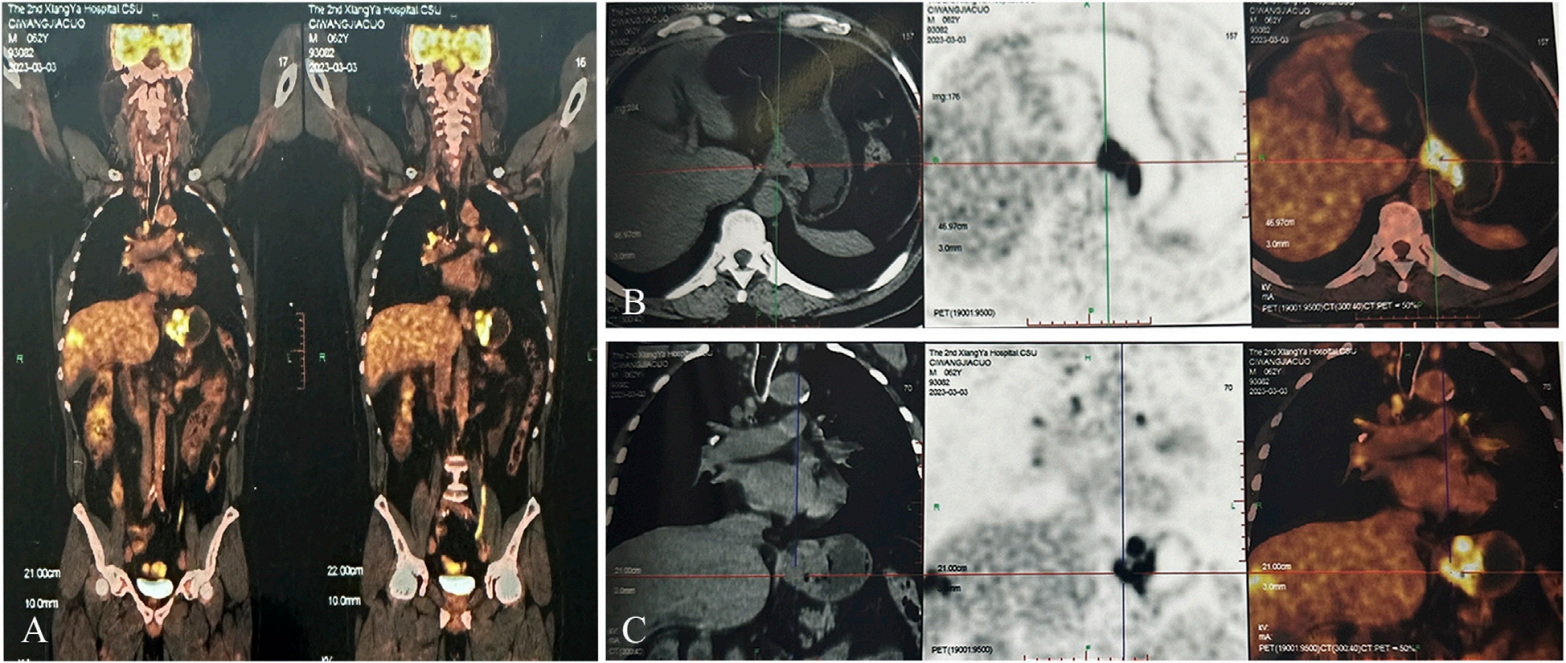
PET-CT examination before initial anti-tumor treatment, considering gastric cardia cancer with peripheral lymph nodes and liver metastasis. **(A)** Overall image. **(B)** Cross-sectional image. **(C)** Coronal image).

**FIGURE 3:**
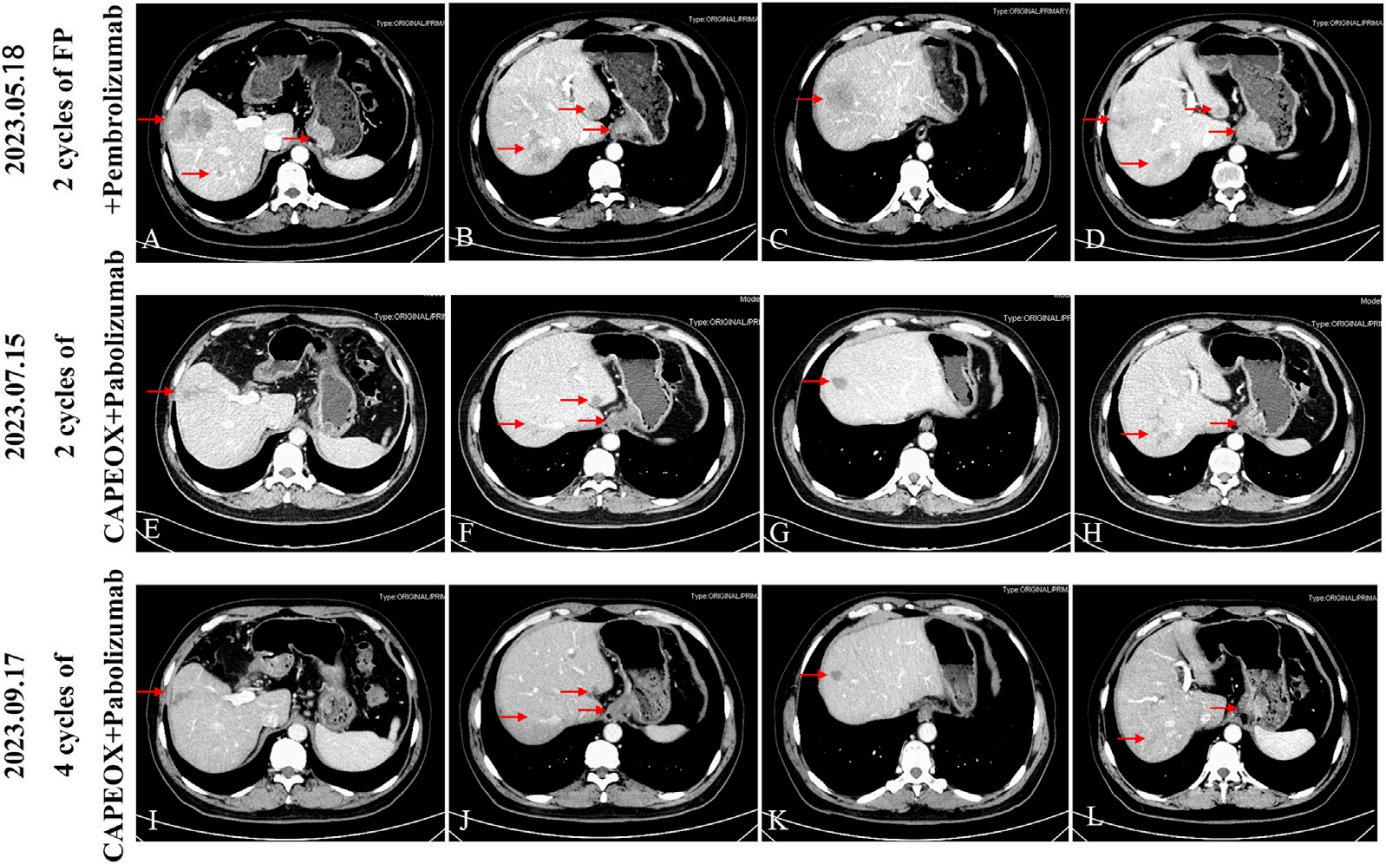
After four cycles of follow-up with CAPEOX combined with pembrolizumab, the efficacy evaluation was sustained partial remission (PR), according to RECIST 1.1. **(A-D)** Progress (PD) after three cycles of FP regimen combined with parolizumab, follow-up CT scan upon admission to our hospital. **(E-H)** After receiving CAPEOX combined with pembrolizumab treatment for two cycles in our hospital, the efficacy evaluation was partial remission (PR). **(I-L)** Four cycles CAPEOX combined with pembrolizumab treatment, the efficacy evaluation was sustained partial remission (PR).

**FIGURE 4:**
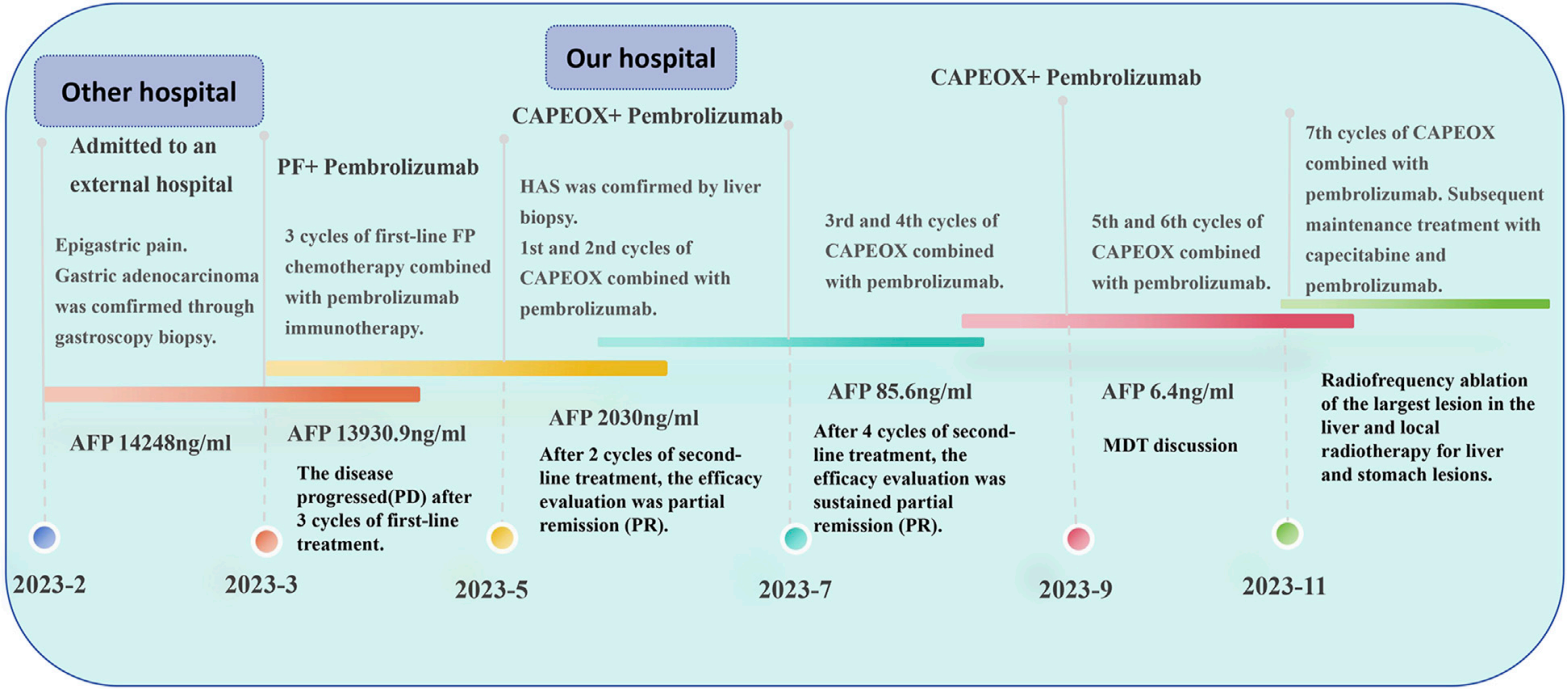
The timeline for patient treatment.

A 62-year-old male was referred to an external hospital complaining with epigastric pain for over 1 month on 27 Feb 2023. The patient had a history of hypertension for 5 years and has been taking irbesartan to control blood pressure. He had no history of hepatitis B virus infection or family history of malignant tumors. Physical examination showed mild tenderness in the upper abdomen. Laboratory examination revealed remarkably increased serum AFP level (14248 ng/mL), whereas other serum tumor markers including CA125, CA199, CEA, and CA724 were within the normal range. An irregular proliferative protrusion and growth mass were observed 37 cm away from the incisors near the entrance of the cardia, indicating obvious mucosal ulceration and involvement of cardiac orifice under gastroscopy. Subsequent abdominal contrast-enhanced CT showed a definite enhanced nodular mass in the cardia and fundus of the stomach, enlarged peripheral lymph nodes, and multiple masses with rim enhancement in liver (Figures 1A–C). No obvious abnormalities were found in thoracic CT scan. Positron emission tomography (PET)-CT confirmed significantly increased glucose metabolism in stomach mass (maximum standard uptake value (SUVmax) = 11.9), peripheral lymph nodes (SUVmax = 8.9) and liver lesions (SUVmax = 8.9) (Figure 2). Pathological examination of cardia neoplasm suggests moderately differentiated adenocarcinoma, with immunohistochemistry stainingHER-2 (+),PD-L1(CPS 20),MLH1 (+),MSH2(+),PMS2(+),MSH6(+). Considering high expression of PD-L1, the patient received first-line FP chemotherapy combined with pembrolizumab immunotherapy at external hospital (fluorouracil 800 mg/m^2^ on day 1 to day 3 + cisplatin 80 mg/m^2^ on day 1 + pembrolizumab 200 mg on day 1, intravenously administered every 3 weeks). The patient tolerated immuno-chemotherapy very well. Unfornately, on April 27 th, 2023, CT scan after three cycles of first-line treatment showed significantly larger and new tumor lesions in liver (Figure 1). Laboratory examination shows no significant decrease in serum AFP level (Figure 4). The disease progressed based on RECIST 1.1. Subsequently, the patient sought further treatment at our hospital. Considering the significant increase in intrahepatic lesions and AFP compared to before, we performed CT guided liver biopsy on the patient. Immunohistochemistry suggested CK7 (−), CK19 (+), CK20 (−), CDX-2 (+), SATB2 (+), TTF-1 (−), SALL4 (+), SOX2 (−), AFP (−), Heppar-1 (−), GPC3 (+), GS (+), CD56 (−), Syn (−), CgA (−), PAX8 (−), PSA (−), GATA3 (−), DPC4 (±) and Ki-67 (+, 50–70%). The final diagnosis support was low differentiated adenocarcinoma, combined with clinical history, histological morphology, and immunohistochemistry, the first consideration was gastric hepatoid adenocarcinoma metastasis. Owing to the patient’s unwillingness of hair loss, which is an adverse reaction of paclitaxel, we prescribed second-line treatment of CAPEOX chemotherapy combined with pembrolizumab (oxaliplatin 130 mg/m^2^ on day 1+ capecitatine 1000 mg/m^2^ twice a day on day 1 to day 14 + pembrolizumab 200 mg on day1, repeated every 3 weeks). Amazingly, the gastric tumor and liver lesions remarkably shrinked after two cycles of treatment on CT scan. And later follow-up CT scan after four cycles showed continuous shrink of primary and metastatic tumors (Figure 3). Serum AFP level decreased significantly (Figure 4). According to RECIST 1.1, the efficacy evaluation was sustained partial remission (PR). After seven cycles of second-line CAPEOX combined with pembrolizumab, the patient underwent MDT discussion and was treated with radiofrequency ablation of the largest lesion in the liver and local radiotherapy for liver and stomach lesions, while maintaining treatment with capecitabine and pembrolizumab. The patient’s second-line progression-free-survival (PFS) is greater than 9 months. The schedule of patient treatment is shown in Figure 4.

## Discussion

HAS is a special subtype of gastric cancer, with a low incidence, easy metastasis in the early stage, and poor prognosis (Zhou et al., 2020). The biggest characteristic of this type of patient is that it can be combined with elevated serum AFP(2, 7, 8). Since 1985, Ishikura et al. (Ishikura et al., 1985) reported a case of pathological differentiation of both adenocarcinoma and hepatocellular carcinoma, and first proposed the concept of HAS. Gastric cancer with differentiated liver cells in pathological morphology can be diagnosed as HAS regardless of whether serum AFP or AFP staining is positive. Its histological characteristics are myeloid, characterized by the pleomorphism of tumor cells and the appearance of hepatocellular carcinoma like differentiation (Ishikura et al., 1997; Nagai et al., 1993; Wang et al., 2019). The morphological characteristics and arrangement of tumor cells are similar to those of liver cancer cells, with large and irregular cells arranged in a beam like or nest like manner, abundant blood sinuses, large nucleoli in the center, visible mitotic figures, meganuclei or deformed nuclei, and bile like substances around tumor cells. Immunohistochemical markers such as Hep Par-1, Glypican-3, SALL4, AFP, CK19, CDX-2 are often expressed, which can assist in diagnosis and exclusion. Studies have shown that tumor markers such as AFP, CEA, and CA199 are not only related to the diagnosis of HAS, but also to the prognosis of patients. Higher tumor markers indicate poorer prognosis in HAS patients, usually (Yang et al., 2023). Another clinical feature of HAS is the high rate of liver metastasis. The patient we reported was discovered multiple liver metastases during the same period. The prognosis of HAS is generally worse than that of ordinary gastric cancer, with a higher rate of vascular lymphatic infiltration and lymph node metastasis, especially liver metastasis, which is as high as 75.6%. Liver metastasis is the main cause of death in HAS patients (Chang et al., 1990). Most research suggests that the key to the treatment of HAS lies in the combination of radical surgical resection and subsequent systemic chemotherapy. Based on domestic and international research results, there are currently multiple chemotherapy regimens such as FLEP regimen, paclitaxel combined with fluorouracil, paclitaxel combined with teziol, paclitaxel regimen, and so on. Although there is no recommended standard chemotherapy regimen, adjuvant chemotherapy has been proven to be an independent factor for long-term survival (Nakao et al., 2015; Zeng et al., 2018; Zhang et al., 2011; Velut et al., 2014; Xiao et al., 2015; Liu et al., 2015). When patients have liver metastasis, interventional therapy with relatively small toxic side effects can be used. In the case of chemotherapy resistance, targeted therapy can be used (Doi et al., 2018). There is no large-scale prospective study, and the treatment of advanced HAS relys on advanced gastric adenocarcinoma, which first-line standard treatment is chemotherapy combined with anti-programmed cell death 1 (PD-1) immunotherapy (Ajani et al., 2022; Smyth et al., 2020). Zhou et al. (Zhou et al., 2023) reported a locally advanced HAS patient who achieved complete pathological response after neoadjuvant chemotherapy combined with immunotherapy. However, this patient had PD-L1 CPS<1, belonging to a subtype that is not sensitive to immunotherapy. This case confirmed the complexity of the HAS immune microenvironment. Although retrospective reports and small sample cases of HAS have been reported in recent years, our understanding of HAS is still very shallow.

Immunotherapy combined with chemotherapy has become the recommended standard first-line treatment for advanced gastric cancer. The KEYNOTE-062 study was a randomized, controlled, partially blinded, phase III trial that evaluated pembrolizumab, pembrolizumab-chemotherapy, and chemotherapy alone as first-line treatment in patients with advanced gastric/gastroesophageal junction (G/GEJ) adenocarcinoma. This study indicates that in the population with PD-L1 CPS ≥1, the combination of pembrolizumab and FP(fluorouracil + cisplatin) regimen chemotherapy is not inferior to FP chemotherapy alone, and there is no significant difference in OS between the two groups (Shitara et al., 2020). CheckMate-649 is a large, phase III randomized, multicenter clinical trial involving 1,581 patients with previously untreated, unresectable, human epidermal growth factor receptor 2 (HER2)-negative G/GEJ. This clinical trial found that regardless of PD-L1 expression level, the combination of nivolizumab and XELOX (oxaliplatin + xeloda) regimen was significantly superior to XELOX chemotherapy alone. The great differentiator between the chemotherapy regimens used in these two studies lies in the platinum class, one is cisplatin and the other is oxaliplatin. Currently, the NCCN guidelines recommend oxaliplatin as the preferred chemotherapy regimen for gastric cancer, rather than cisplatin (Janjigian et al., 2021). The Keynote-859 study released at the American Society of Clinical Oncology (ASCO) conference in 2023 showed that regardless of PD-L1 expression level, the combination of pembrolizumab and chemotherapy was significantly superior to chemotherapy alone. However, more than 86% of patients with chemotherapy regimen were treated with CAPEOX, and a few patients were treated with FP regimen. In subgroup analysis, the combination of FP regimen and pembrolizumab was not superior to chemotherapy alone, while the combination of CAPEOX regimen and pembrolizumab was superior to chemotherapy alone (Rha et al., 2023). Studies have shown that oxaliplatin has a synergistic effect with PD-1 monoclonal antibody, which may be the reason why the FP regimen combined with pembrolizumab is not superior to chemotherapy alone.

Immunotherapy represented by immune checkpoint blockade (ICB) has been widely used in clinical practice, but the response rate is limited due to suppressive tumor immune microenvironment (TIME) and insufficient tumor immunogenicity. Chemoimmunotherapy is becoming one of the best combination strategies for improving ICB response. Research has found that oxaliplatin based drugs can induce immunogenic cell death, restore the tumor immune microenvironment, and promote the development of the tumor immune microenvironment towards in tumor clearance direction. When used in combination with PD-1, tumor infiltration of Tregs is significantly reduced, and the CD8 T/Treg ratio increases (Hao et al., 2023), thereby avoiding the immune desert phenotype, which explains the effective synergy of immunogenic chemotherapeutic drugs and ICB. So far, apart from microsatellite instability-high (MSI-H) and tumor mutational burden high (TMB-H), there are no very precise biomarkers for predicting the efficacy of immune checkpoint inhibitors or resistance to immune checkpoint inhibitors (Guven et al., 2022; Viscardi et al., 2022).

Based on the molecular characteristics of our reported case of hepatoid adenocarcinoma (Her-2 is negative, PD-L1 is strongly positive), the first-line combination of pembrolizumab and FP regimen chemotherapy progressed rapidly, while the second-line combination of parolizumab and CAPEOX regimen achieved good efficacy, achieving partial relief and significant clinical symptom relief, consistent with current clinical trial results. The different results obtained by the two regimens may be related to the synergistic effect of oxaliplatin and immunotherapy. There is currently no standard treatment plan for HAS, and this pathological report provides some new clues for the treatment of HAS.

## Conclusion

In summary, this study reported a rare case of HAS where the first-line PF regimen combined with pembrolizumab showed poor efficacy, while the second-line CAPEOX regimen combined with pembrolizumab immunotherapy achieved partial remission. Oxaliplatin combined with immunotherapy may enhance the efficacy of anti-tumor therapy by inducing immunogenic cell death, enhancing the lytic effect of cytotoxic T lymphocytes (CTL) on tumor cells, and regulating the tumor microenvironment. The selection of chemotherapy regimen and immunotherapy provides a new perspective. Further research is necessary to explore the potential mechanisms of chemotherapy combined with immunotherapy, providing some reference for the treatment of HAS. Close follow-up observation of adverse reactions in cancer patients should also be conducted during the immunotherapy process.

## Data Availability

The raw data supporting the conclusions of this article will be made available by the authors, without undue reservation.
